# Topics of Public Interest

**Published:** 1911-07

**Authors:** 


### TOPICS OF PUBLIC INTEREST

### Acute Epidemic Poliomyelitis

THE following circular has been issued by the American Orthopedic Association and the American Pediatric Society in reference to acute epidemic polyomelitis and addressed to health authorities and boards of health:

Anterior poliomyelitis is, so far as known, a communicable disease, being communicated from one patient to another and also by means of a third person. It occurs in epidemics and tends to spread along the lines of greatest travel. There is reason to believe that it is prevented from spreading by quarantine, and with the very great prevalence of the disease in the summer of 1910 it is the opinion of this committee that it is essential that it should be made a reportable disease in all states in order that its presence may be detected and its spread guarded against.

Of particular significance are the so-called abortive cases, where indefinite ailments occur in children in communities where frank paralysis also exists. These abortive cases of infantile paralysis are undoubtedly a source of infection, and their record and study is of much importance. In a community where cases of infantile paralysis occur, cases of illness with sudden onset of fever and meningeal symptoms should be closely watched and regarded as possibly infectious. In such cases even recovery without paralysis does not establish the fact, that the case was not abortive infantile paralysis.

All cases of infantile paralysis should be strictly quarantined, sputum, urine and feces being disinfected, and the same rigid precautions being adopted as in scarlet fever. This quarantine should, in the opinion of the committee, last for four weeks in the absence of definite knowledge as to when the infection ends. Children from infected families should not be allowed to go to school until the quarantine is abandoned. The transportation or transfer of acute cases in public conveyances should be strictly forbidden. It would be very desirable to adopt provisional quarantine measures in suspicious cases in a community where an epidemic prevails. The report of all cases of infantile paralysis to the public health authorities should be enforced by law, and all deaths from this cause should be properly described and registered. A careful study of epidemics by public health authorities is strongly advised.

ROBERT W. LOVETT, M. D., Chairman,
HENRY KOPLIK, M. D.,
H. WINNETT ORR, M. D.,
IRVING M. SNOW, M. D., Secretary.

### Open Air Schools

THE park commissioners have selected a site for the out-of-door vacation school that is to be maintained this summer by the Teachers' Educational League. It is near the Delaware avenue entrance, the old water gate of the Pan-American days, and close to the shelter-house. It will be opened on July 10 to be continued for six weeks, six days a week. There will be 40 children accommodated who are to be chosen by the principals of the public schools. The out-of-door school is for children between seven and twelve who are not robust. The daily sessions will begin at 9 a. m., and last until 5 p. m. The studies will be mainly nature studies, with a little book instruction. There will be a luncheon served the children in the morning followed by a good dinner at noon and a light luncheon before they are dismissed for the day. Miss Clara Bredell will be in charge, assisted by teachers from the league.

There will be three large tents pitched, a dining tent, a kitchen tent and a large shelter tent for the school work when it rains. The project has the support of Superintendent Emerson and is endorsed by Dr. F. E. Fronczak, the health commissioner of Buffalo.

### Cold Storage Bill Signed—State Regulation of All Plants

STATE regulation of cold storage plants is provided for in the bill of Assemblyman Brennan, which was signed by Governor Dix June 15, 1911.

The new law, which goes into effect immediately, provides that all cold storage goods shall be so marked and that they shall not be kept in storage for more than ten months, except butter products, which may be held twelve months. The State Health Department has authority to inspect and supervise all cold storage plants and to make reasonable rules and regulations governing them. Warehousemen are required to file a report to the State Health Department in January, May and September in each year, setting forth the quantity of food stuffs in cold storage. The act prohibits the return of food to cold storage when once placed on the market for sale.

Violation of the new law is made a misdemeanor.

### Fourth International School Hygiene Congress

A SUBCOMMITTEE has been appointed to draw up a plan for financing the congress when it convenes in Buffalo, in August, 1913, and for entertaining the delegates. It is expected that about $10,000 will be needed, which will probably be raised by private subscription.

Those named on the subcommittee are as follows: Superintendent Henry P. Emerson, representing the department of public instruction; commissioner Francis E. Fronczak, representing the health department; City Clerk Harold J. Balliett, who is to act as secretary of the committee; the Reverend Edmund Gibbons, representing the private schools; Mrs. John Miller Horton, representing the women's clubs; T. Guilford Smith, representing the citizens at large; A. H. Whitford, representing the Chamber of Commerce; Dr. D. V. McClure, representing the physicians.

Dr. Francis E. Fronczak, health commissioner of Buffalo, and who succeeded in having the congress convene here by attending the meeting held in Paris a year ago was appointed chairman of the committee.

---

### Single Medical Examining Board for Pennsylvania

GOVERNOR TENER has signed the one-board medical bill passed at the session of the Pennsylvania Legislature just closed. The new law provides for the establishment of a Bureau of Medical Education and Licensure, consisting of seven members, the superintendent of Public Instruction and the Commission of Health being members *ex officio* and the remaining five to be appointed by the Governor and to be selected from the three present legally incorporated medical societies of the state—one from the Medical Society of the State of Pennsylvania, one from the Homeopathic, and one from the Ecletic Society— who shall be licensed and qualified to practise under existing laws and shall have practised not less than ten years in the state; the two remaining members shall not both be of the same school or system of practice. No member of the bureau shall be a member of the faculty of any school, college, or university teaching medicine or surgery. In addition to medical licensure the board shall have the duty of ascertaining and reporting annually the character of the instruction given by each medical institution chartered in the State and its facilities for teaching the various departments of medicine in accordance with the standard required by the act. This comprises a general preliminary education of not less than a standard four years' high school course or its equivalent and a graded medical and surgical course of four years, each of which shall be not less than 35 hours each week of active work in didactic, laboratory and clinical study in different calendar years.

Any medical institution chartered by the state and empowered to grant the degree in medicine that shall be unanimously adjudged by the bureau as failing to maintain the required standard shall be duly notified, and failure to conform after notification shall render graduates of that institution ineligible for license. The bureau is further empowered to examine any persons pretending to a knowledge of a minor subject or subjects pertaining to medicine and surgery or semimedical and surgical subjects who have schools and colleges teaching such subjects, for the purpose of establishing regulation and state licensure. The bureau is to have the same kind of oversight over such schools as over medical schools, and it is to conduct such limited examinations as may be necessary to determine whether candidates have the requisite degree of knowledge of their subject and are otherwise worthy of state licensure. After a system of special licensure shall have been established it will be unlawful to practise in any of these special pursuits without a license, and the general provisions of the act become applicable to the special practitioner. The new act does not affect the practice of dentistry, pharmacy, and osteopathy, for each of which provision is made by existing laws.

---

Appendicitis in Childhood.—In a paper based on the analysis of 500 cases, H. C. Deaver, Philadelphia (*Journal A. M. A.*, December 24), insists on the importance and frequency of appendicitis in childhood. The number of cases increases in frequency with age from birth to puberty, and it is more common in males. There is less tendency to formation of strictures, but fecal concretions are more frequent. As predisposing causes he mentions enteric fever, intestinal catarrh and influenza, but other infections and nasopharyngeal trouble are hardly to be considered as etiologic factors. The symptoms in infants are often scanty, irregular and misleading, and the disease is more frequent than the statistics show. In older children acute attacks occur suddenly with stormy symptoms even more frequently than in adults. Chronic appendicitis indicates a focus of chronic autotoxemia with all its bad results. With early diagnosis and operation the prognosis is favorable, but it rapidly becomes worse after twenty-four hours. Non-operative treatment is indicated in localised abscess with diffuse peritonitis. Opium and purgatives are absolutely contraindicated. Operation is even more suitable to children than to adults and post-operative treatment is very important. The Fowler position must not be kept up for more than thirty-six hours in drainage cases, lest intestinal obstruction be set up. The symptoms of obstruction are sudden severe pain, becoming paroxysmal and by a nausea, spitting up and vomiting. With numerous adhesions ileocolostomy is the best procedure. Drainage is to be employed only when the exudate is purulent or profuse. It produces adhesions and predisposes to intestinal obstruction. A wet dressing is the best for absorption.

